# Angiotensin-Induced Abdominal Aortic Aneurysms in Hypercholesterolemic Mice: Role of Serum Cholesterol and Temporal Effects of Exposure

**DOI:** 10.1371/journal.pone.0084517

**Published:** 2014-01-23

**Authors:** Petra A. Prins, Michael F. Hill, David Airey, Sam Nwosu, Prudhvidhar R. Perati, Hagai Tavori, MacRae F. Linton, Valentina Kon, Sergio Fazio, Uchechukwu K. Sampson

**Affiliations:** 1 Department of Medicine, Vanderbilt University Medical Center (VUMC), Nashville, Tennessee, United States of America; 2 Department of Biostatistics, VUMC, Nashville, Tennessee, United States of America; 3 NCI Information Systems, Inc. Nashville Tennessee, United States of America; 4 Department of Pediatrics, VUMC, Nashville, Tennessee, United States of America; 5 Department of Pathology, Microbiology and Immunology, VUMC, Nashville, Tennessee, United States of America; 6 Department of Radiology and Radiological Sciences, VUMC, Nashville, Tennessee, United States of America; Max-Delbrück Center for Molecular Medicine (MDC), Germany

## Abstract

**Objective:**

Understanding variations in size and pattern of development of angiotensin II (Ang II)-induced abdominal aortic aneurysms (AAA) may inform translational research strategies. Thus, we sought insight into the temporal evolution of AAA in apolipoprotein (apo)E^−/−^ mice.

**Approach:**

A cohort of mice underwent a 4-week pump-mediated infusion of saline (n = 23) or 1500 ng/kg/min of Ang II (n = 85) and AAA development was tracked via *in vivo* ultrasound imaging. We adjusted for hemodynamic covariates in the regression models for AAA occurrence in relation to time.

**Results:**

The overall effect of time was statistically significant (p<0.001). Compared to day 7 of AngII infusion, there was no decrease in the log odds of AAA occurrence by day 14 (−0.234, p = 0.65), but compared to day 21 and 28, the log odds decreased by 9.07 (p<0.001) and 2.35 (p = 0.04), respectively. Hemodynamic parameters were not predictive of change in aortic diameter (Δ) (SBP, p = 0.66; DBP, p = 0.66). Mean total cholesterol (TC) was higher among mice with large versus small AAA (601 vs. 422 mg/ml, p<0.0001), and the difference was due to LDL. AngII exposure was associated with 0.43 mm (95% CI, 0.27 to 0.61, p<0.0001) increase in aortic diameter; and a 100 mg/dl increase in mean final cholesterol level was associated with a 12% (95% CI, 5.68 to 18.23, p<0.0001) increase in aortic diameter. Baseline cholesterol was not associated with change in aortic diameter (p = 0.86).

**Conclusions:**

These are the first formal estimates of a consistent pattern of Ang II-induced AAA development. The odds of AAA occurrence diminish after the second week of Ang II infusion, and TC is independently associated with AAA size.

## Introduction

Angiotensin II (AngII)-induced models of abdominal aortic aneurysms (AAA) constitute the bedrock of experimental strategies to elucidate the pathobiology of AAA and for the development of therapeutic interventions. Sustained subcutaneous infusion of AngII causes the development of AAA in genetically engineered mice expressing spontaneous or diet-induced hypercholesterolemia [1]–[7]. The infusion of 500 to 1500 ng/kg/min of AngII results in AAA in up to 100% of exposed animals [2], [8]–[9]. Although the AngII-induced model shares similarities with the human disease, the incidence of experimental AAA is reportedly independent of blood pressure and is augmented by hyperlipidemia [2]. Despite widespread employment of this model for the interrogation of the pathobiology of AAA disease processes, there is no apparent reason for the observed diversity in AAA size noted in this model despite controlled experimental conditions [9], nor is there a well-characterized pattern of development. In this context, it is not clear whether systemic hemodynamic conditions modulate differences in AAA size and temporal evolution. Similarly, although hypercholesterolemia is known to augment the incidence of AAA, the relationship between the degree of hypercholesterolemia and the temporal evolution and size of AAA is unknown.

Overall, a better understanding of these relationships, and a detailed characterization of the pattern of AngII-induced AAA development may help optimize translational research strategies. Consequently, we employed noninvasive *in vivo* ultrasound (US) imaging technology to assess the temporal evolution of AngII-induced AAA in hypercholesterolemic mice, and to evaluate the potential role of systemic hemodynamic conditions and serum total cholesterol in the heterogeneity of AAA sizes. Herein we report the first formal estimates of the odds of AAA occurrence vis-à-vis time in the context of a consistent pattern of AngII-induced AAA development in hypercholesterolemic mice. Furthermore, we demonstrate that the extent of hypercholesterolemia is associated with aneurysmal size, a finding that has potential clinical relevance for the management of AAA patients.

## Methods

### Mice

A total of 108 male ApoE^−/−^ mice on C57BL/6 background were purchased from Jackson Laboratories (JAX® Mice and Services, Bar Harbor, Maine USA) and maintained on normal mice chow diet (RP5015; PMI Feeds Inc.). Animal care and experimental procedures were carried out according to the regulations of the Division of Animal Care at Vanderbilt University and were approved by the Institutional Animal Care and Usage Committee.

### Ultrasound imaging, AngII infusion, and determination of aortic diameters

Animal preparation: Mice were anesthetized in a chamber using isoflurane (2–2.5% for induction, 1.25–1.75% for maintenance). Anesthetized mice were then secured in a supine position onto a mouse-handling platform with temperature and heart rate monitoring electrodes. Secured mice were shaved from the thoracic to the lower abdominal region using a depilatory cream (Nair; Church & Dwight Co., Inc., Princeton, NJ). A pre-warmed US transmission gel (Aquasonic 100; Parker Laboratories, Orange, NJ) was applied onto the shaved region to facilitate transmission of US signals. Image acquisition: We employed a high-resolution US imaging system (Vevo-770, VisualSonics, Toronto, Canada). Mice underwent a 6–7 hour fast before repeated B-Mode (2-D) short axis (SAX) and long axis (LAX) cine loops of the aorta were acquired using a real-time micro visualization scan head (RMV-704 transducer) at a central frequency of 40 MHz, a focal length of 6 mm, 10×10 mm field of view, and a frame rate of 34 Hz.

After baseline imaging, mice were infused with AngII (cat# A9525, Sigma-Aldrich, Inc.) (1500 ng/kg/min) via subcutaneously implanted osmotic mini-pumps (Alzet, model 2004; Durect Corporation, Cupertino, CA) as previously reported [10]. While most studies of AAA in mice have employed the 1000 ng/kg/min dosing regimen for AngII, we have used AngII infusion via mini-pump in hyperlipidemic mice for many years, including 500, 1000, and 1500 ng/kg/min dose regimens [3]. In addition, the initial observation about the distinct pattern of temporal evolution and size was made in experiments that employed the use of 1000 ng/kg/min [8]. The dose of 1500 ng/kg/min causes the highest incidence of AAA in the timeline of our studies, thus allowing a more efficient separation between no aneurysm formation and small or large AAA. .

Mice underwent serial US imaging during AngII infusion to determine the evolution of AAA, and to assess for quantitative regression of AAA following AngII withdrawal. The experiment lasted 4 weeks (28 days of infusion) with the exception of a small subset of mice that where followed an additional number of days after retraction of the subcutaneous pumps after 28 days, and was guided in its entirety by *in vivo* US imaging surveillance every 48 to 72 hours. Mice were sacrificed for *ex vivo* evaluation at the end of the experiment (41 days). All US imaging aortic diameters were determined by measuring between the outer walls of the aorta. 2-D guided Power and spectral Doppler patterns aided target-vessel confirmation. Image analyses were done off-line on digitally stored recorded cine loops at variable frame rates of 1 to 100 Hz using the built-in software analysis package of the Vevo770 US imaging system.

Please see online supplementary methods S1 for details on tail vein injection, hemodynamic assessment, atherosclerotic lesion quantification, quantitative gene expression, analyses of serum total cholesterol, lipoproteins, and PCSK9, zymography for metalloproteinases, and immuno-histology and fluorescence analysis.

### Statistical analyses

Wilcoxon, Kruskal Wallis, ANOVA, and student t tests were used as deemed appropriate for comparison between groups. Box plots were used to represent baseline hemodynamic data according to exposure and outcome, while mean plots were used to illustrate the serial hemodynamic data by exposure and outcome. Linear mixed effects regression analysis was used to assess aortic diameter and pre-specified covariates: time of measurement, AngII exposure, and hemodynamic parameters (SBP, DBP, PP, PR, MAP). The odds of AAA occurrence vis-à-vis duration of Ang II exposure were assessed by mixed logistic regression analysis. A linear mixed effect model was used to assess the relationship between all hemodynamic parameters and delta. Nonparametric and linear regression methods were used to evaluate total cholesterol in relation to changes in AAA diameter. Statistical modeling was conducted using R statistical package [11].

## Results

**Figure 1:**
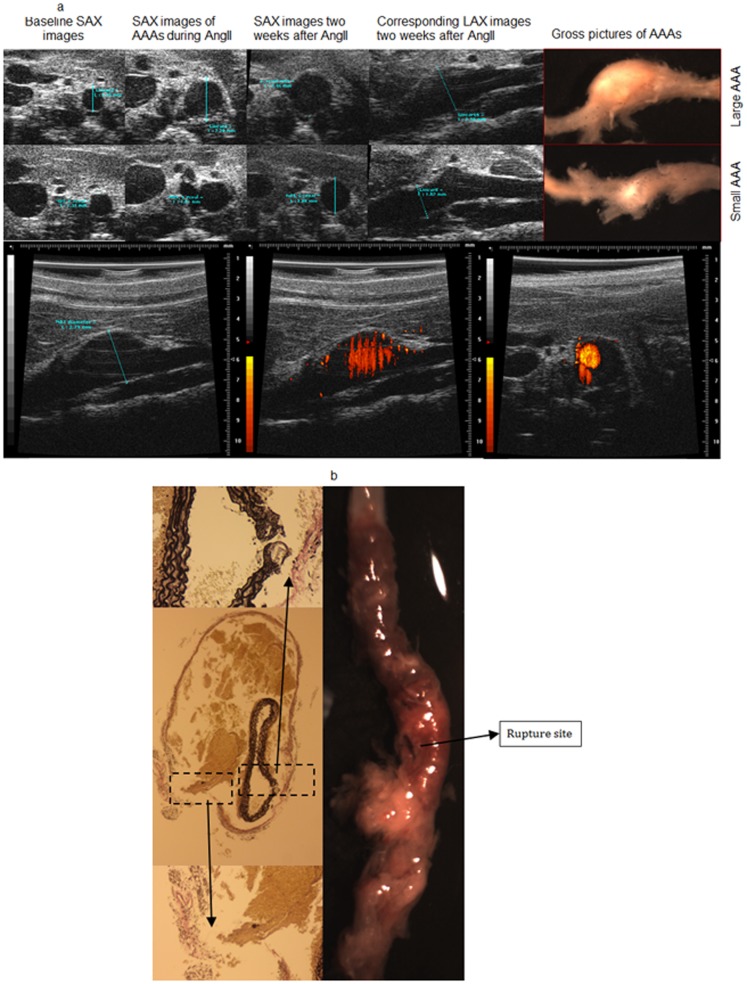
Representative images of AAA. (A) Representative *in vivo* US and gross anatomic images of intact AAA. Top panels: Representative SAX and LAX images with corresponding gross anatomical pictures of a large and small AAA, respectively. Bottom: Representative US Power Doppler imaging technique employed for target-vessel confirmation. Power Doppler images delineate blood flow in a large AAA in LAX and SAX views. US, ultrasound; SAX, short axis; LAX, long axis; Ang II, angiotensin II; AAA, abdominal aortic aneurysm. (B): Representative image of gross anatomic and histological section of a ruptured AAA. Left: Middle panel is an elastin stained cross section demonstrating elastin and adventitial disruption of an AAA; the top and bottom panels are magnified areas of elastin and adventitial break, respectively. Right: Gross image of an abdominal aorta demonstrating a ruptured aneurysm. AAA, abdominal aortic aneurysm.

Of the 108 mice studied, 85 underwent AngII infusion while 23 received saline infusion. In the AngII group, 72 mice (85%) developed AAA, of which 55 were intact and 17 were ruptured. Thirty-one (56%) of the intact AAA were small (1.2– 1.5× baseline diameter and 24 (34%) were large (≥1.5× baseline diameter). There was no incidence of AAA in the saline group. Figure 1a illustrates representative long and short axis *in vivo* US images of AAA with corresponding gross anatomic pictures; similarly, a representative image of a ruptured aneurysm is depicted in Figure 1b.

### Pattern of AAA Evolution

**Figure 2:**
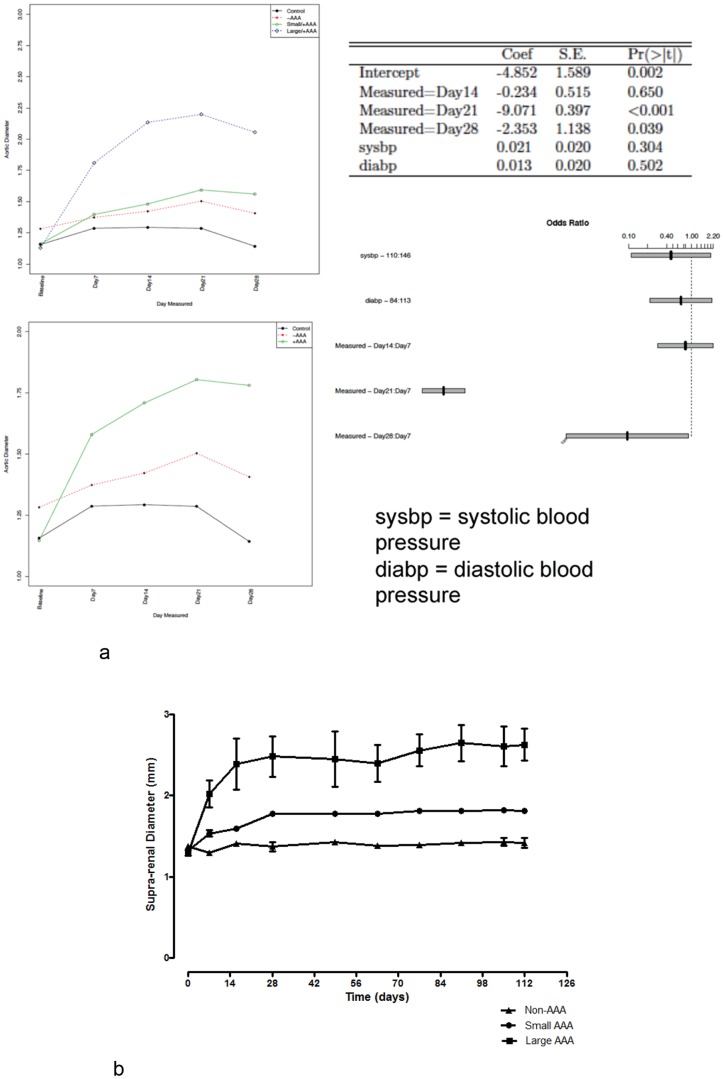
Pattern of AAA development (A) Consistent Temporal Pattern of AAA Development. Temporal evolution of AAA demonstrating the earlier occurrence of large aneurysms in contradistinction to the later development of small aneurysms in mice that underwent continuous 4-week infusion of Ang II (n = 85). The size phenotype appears to be defined at the outset of AAA development, thus suggesting differential susceptibility to Ang II-induced AAA development in genetically homogenous mice. (B): No Quantitative AAA regression Following Cessation of Angiotensin. Continued *in vivo* US surveillance of a subgroup of mice (n = 10) for 90 days post Ang II infusion did not reveal any evidence of quantitative change in the size of either small or large AAA.

An evaluation of the temporal evolution of AngII-induced AAA (Figure 2a) reveals that the occurrence of large aneurysms was prompt, within 3–8 days after initiation of infusion. Of note, over 90% of ruptured AAA leading to death also occurred within 8 days of AngII infusion. Subsequent aneurysms were small and evolved in the second week of AngII infusion. The overall effect of time was statistically significant (p<0.001) (Figure 2a). When compared to day 7 of the study period, there was no decrease in the log odds of AAA occurrence by day 14 (−0.234, p = 0.65). However, compared to 21 and 28 days of AngII infusion, the log odds of an AAA occurrence decreased by 9.07 (p<0.001) and 2.35 (p = 0.04), respectively (Figure 2a). Overall, the degree of initial dilatation of suprarenal aorta in response to AngII portended the final size of the AAA. An analysis of changes in diameter by category of AAA size demonstrated no statistically significant (p = 0.1) quantitative change in diameter regardless of AAA size, 90 days following termination of the 4-week infusion of AngII (Figure 2b).

### Hemodynamics and AAA

**Figure 3:**
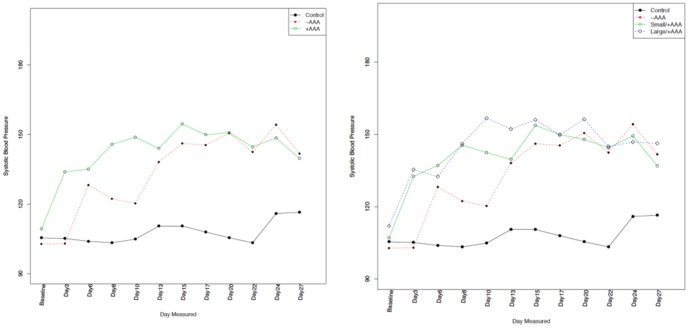
Systemic Hemodynamics and AAA development. Left panel, serial hemodynamic assessment (n = 59) demonstrates an increase in systolic blood pressure following Ang II infusion, which plateaued in the second week of infusion. However, among mice exposed to Ang II there were no differences in systolic blood pressure between those that developed AAA and those that did not. Right panel, similar systolic blood pressure pattern was noted independent of the size of AAA. Overall, statistical comparison revealed no significant difference in all hemodynamic parameters between mice that developed AAA versus those that did not; likewise, there was no difference by AAA size.

Among the mice (n = 59) that underwent hemodynamic monitoring, there was no difference in baseline aortic diameter (1.156±0.089 vs. 1.180±0.111 mm, p = 0.48), systolic blood pressure (SBP) (105.4±9.5 vs. 109.0±8.9 mmHg, p = 0:24), diastolic blood pressure (DBP) (77.7±11.9 vs. 82.3±9.2 mmHg, p = 0.22), mean arterial pressure (MAP) (87.1±10.9 vs. 91.3±8.7 mmHg, p = 0.20), pulse pressure (PP) (27.7±6.0 vs. 26.7±6.3 mmHg, p = 0.47), or pulse rate (548±53 vs. 547±46 beats per minute, p = 0.8). Table 1 provides the complete distribution of baseline hemodynamic parameters. During the 4-week AngII infusion period, there were no differences in SBP measurements by absence, presence, or size of AAA (Figure 3); similar findings were noted in the analysis of DBP, MAP, PP and PR measurements (supplementary Figure 1a to 1d). In our mixed effects model, none of the hemodynamic parameters were predictive of change in aortic diameter from baseline (SBP, p = 0.66; DBP, p = 0.66; MAP, p = 0.55; PP, p = 0.66; and PR, p = 0.39).

**Table 1:** Baseline Summary Statistics.

	ControlN = 13	AngIIN = 46	Test Statistic
Aortic Diameter	1.070 1.150 1.250 (1.156±0.089)	1.080 1.170 1.270 (1.180±0.111)	F_1.57_ = 0.51, P = 0.48
Systolic BP	98.9 107.8 107.9 (105.4±9.5)	103.3 108.5 113.6 (109.0±8.9)	F_1.57_ = 1.4, P = 0.24
Diastolic BP	70.6 79.8 87.2 (77.7±11.9)	76.6 81.5 88.7 (82.3±9.2)	F_1.57_ = 1.5, P = 0.22
MAP	81.7 89.3 94.3 (87.1±10.9)	86.6 91.3 97.0 (91.3±8.7)	F_1.57_ = 1.7, P = 0.2
Pulse Rate	507 556 568 (548±53)	518 553 580 (547±46)	F_1.57_ = 0.06, P = 0.8
**Pulse Pressure**	**25.3 27.2 29.9 (27.7±6.0)**	**22.7 25.5 29.5 (26.7±6.3)**	**F_1.57_ = 0.53, P = 0.47**

*a* b *c* represent the lower quartile *a*, the median *b*, and the upper quartile *c* for continuous variables. *x* ± *s* represents X ± 1 SD.

Test used: Wilcoxon Test.

Aortic diameter is in millimeters; systolic BP (blood pressure), diastolic BP, MAP (mean arterial pressure) and pulse pressure are measured in mmHg.

### Serum Cholesterol, Atherosclerosis, and AAA

**Figure 4:**
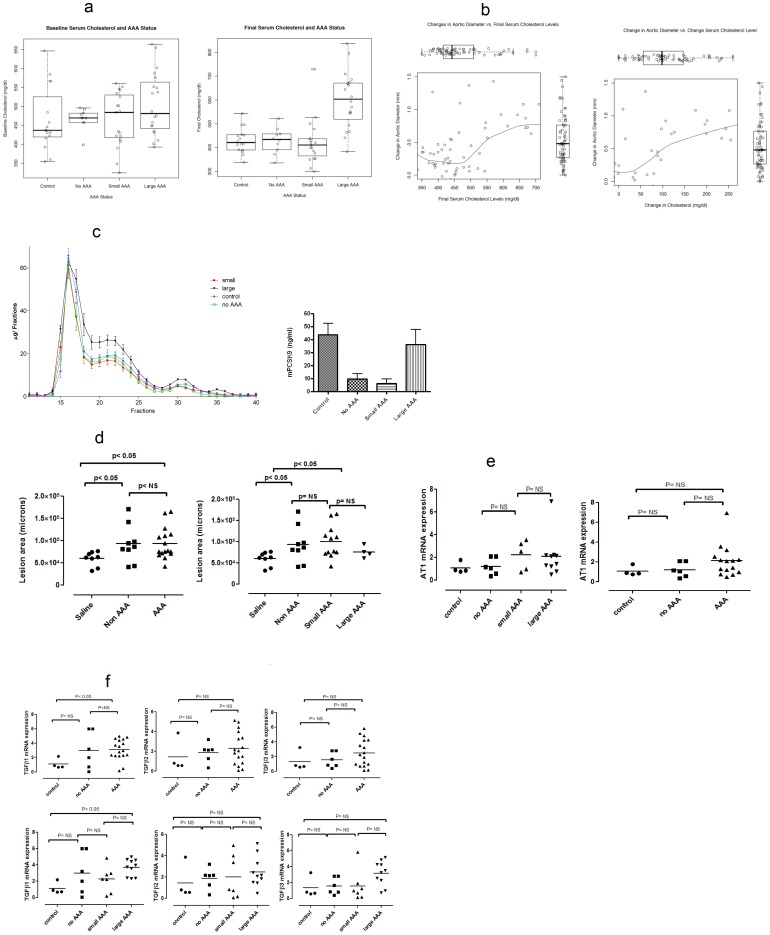
Relationships between total cholesterol, serum lipoproteins and atherosclerosis and AAA. (A) Relationship between total cholesterol concentration (TC) and change (delta) in aortic diameter. Left) Analysis of baseline serum cholesterol of 65 mice revealed no difference in cholesterol levels prior to treatment (control n = 16, no AAA n = 10, small AAA n = 19, and large AAA n = 20; p = 0.25). Right) Final total cholesterol levels, after 4 weeks infusion treatment, prior to sacrifice, revealed statistically significant increased levels of TC in mice that developed large AAA (control n = 16, no AAA n = 10, small AAA n = 19, large AAA n = 20, p<0.0001). (B) Graphical evaluation of both change in aortic diameter versus final cholesterol levels (left), and change in aortic diameter versus change in cholesterol levels (right) reveal clear first order trends suggested by the nonparametric smoothing lines. The separate distribution of each variable is captured the box plot insert opposite its axis. Formal evaluation by regression modeling indicates that both AngII exposure and final cholesterol levels are predictive of change in aortic diameter whereas baseline cholesterol level was not. (C) Serum lipoprotein analysis and PCSK9 serum levels. Left: FPLC analysis of cholesterol profile of each group of mice (control n = 16, no AAA n = 10, small AAA n = 19, large AAA n = 20). There was a statistically significant difference between large AAA and the other groups for LDL (fractions 20–25) (p<0.05). Right: PCSK9 serum levels are significantly reduced by AngII exposure (no AAA and small AAA) except in mice with large AAA, where PCSK9 levels are similar to those of controls (control n = 16, no AAA n = 10, small AAA n = 19, and large AAA n = 20; p = 0.0019). (D) Atherosclerosis and AAA. Left: Mice treated with Ang II demonstrate significant increase in the extent of atherosclerosis in the aortic arch (control n = 8, no AAA n = 9, small AAA n = 12 and large n = 4 p<0.05). No difference was seen between mice that developed AAA and those that did not. Right: Analysis of small and large AAA showed no differences in plaque formation. (E) AT1 expression and AAA. The differences in AT1 expression according Ang II exposure, AAA occurrence, or AAA size were not statistically significant (control n = 4, no AAA n = 6, small AAA n = 5 and large AAA n = 10). (F) TGFß and AAA. Comparison of differences in the expression of TGFβ 1, 2, and 3 between mice with and without AAA, and by AAA size, was statistically significant for TGFβ 1 when controls and AAA were compared (control n = 4, no AAA n = 6, small AAA n = 7 and large AAA n = 10; p<0.05). Analysis of TGFβ1 expression in AAA was significant only for the large AAA versus control.

There were no differences in serum cholesterol at baseline for mice of different groups (p = 0.25; means: control 465 mg/dl, no AAA 467 mg/dl, small AAA 465 mg/dl and large AAA 506 mg/dl) (Figure 4a, left). However, final cholesterol levels differed by exposure status (p = 0.005; means: control 429 mg/dl vs 497 mg/dl in the AngII group). This difference was driven by mice that developed large AAA following AngII exposure: control 429 mg/dl (n = 16), no AAA 429 mg/dl (n = 10), small AAA 422 mg/dl (n = 19), and large AAA 601 mg/dl (n = 20); p<0.0001 (Figure 4a, right). In the graphical evaluation of the relationship between change in aortic diameter and final cholesterol levels (Figure 4b, left) and change in cholesterol (Figure 4b, right), both nonparametric smoothing lines suggest first order relationships. In the adjusted regression model for change in aortic diameter, AngII exposure was associated with 0.43 mm (95% CI, 0.27 to 0.61, p<0.0001) increase in aortic diameter, a 100 mg/dl increase in mean final cholesterol level was associated with a 12% (95% CI, 5.68 to 18.23, p<0.0001) increase in aortic diameter, but baseline cholesterol was not associated with change in aortic diameter (p = 0.86). Similar results were obtained in the analysis of the relationship between change in aortic diameter and the change in cholesterol level (final minus baseline levels); AngII exposure was associated with 0.47 mm (95% CI, 0.30 to 0.65, p<0.0001) increase in aortic diameter and a 100 mg/dl mean change in cholesterol was associated with 8%(95% CI, 3 to 14, p = 0.004).

We studied plasma lipids (FPLC analysis) not as standard lipid panel but as size-based separation of all lipoproteins (Figure 4c). The results shows a significant difference only for the low density lipoprotein (LDL) “shoulder” of the much larger very low density lipoprotein (VLDL) peak typical of these mice on the high-fat diet. What is remarkable is that profiles from all mice are exactly overlapping, except for the large AAA group indicating a significant increase in LDL cholesterol (Figure 4c, left) compared to controls and those with no AAA (p = 0.03). Not only did mice with large AAA statistically differ from controls and those without AAA, the difference between large and small AAA was also significant (p = 0.041). Serum PCSK9 levels were significantly reduced in both the no AAA and small AAA groups compared to control (p = 0.002). In mice with large AAA serum PCSK9 levels were similar to those of control mice (Figure 4c, right).

The burden of atherosclerosis was greater among AngII-exposed mice versus control (Figure 4d); however, there was no difference in the extent of atherosclerosis regardless of the occurrence or size of AAA (Figure 4d). Of note, there was no difference in the degree of AT1 receptor gene expression (Figure 4e, left panel) according to AngII exposure; similarly, AT1 receptor gene expression did not differ by size of AAA (Figure 4e, right panel). TGFß 1 expression was significantly higher in AngII infused mice than in controls, and the difference was mostly due to the mice with large AAA (p<0.05). No differences in TGFß 2 and 3 levels were seen control and AngII infused mice (Figure 4f).

### Macrophages

**Figure 5:**
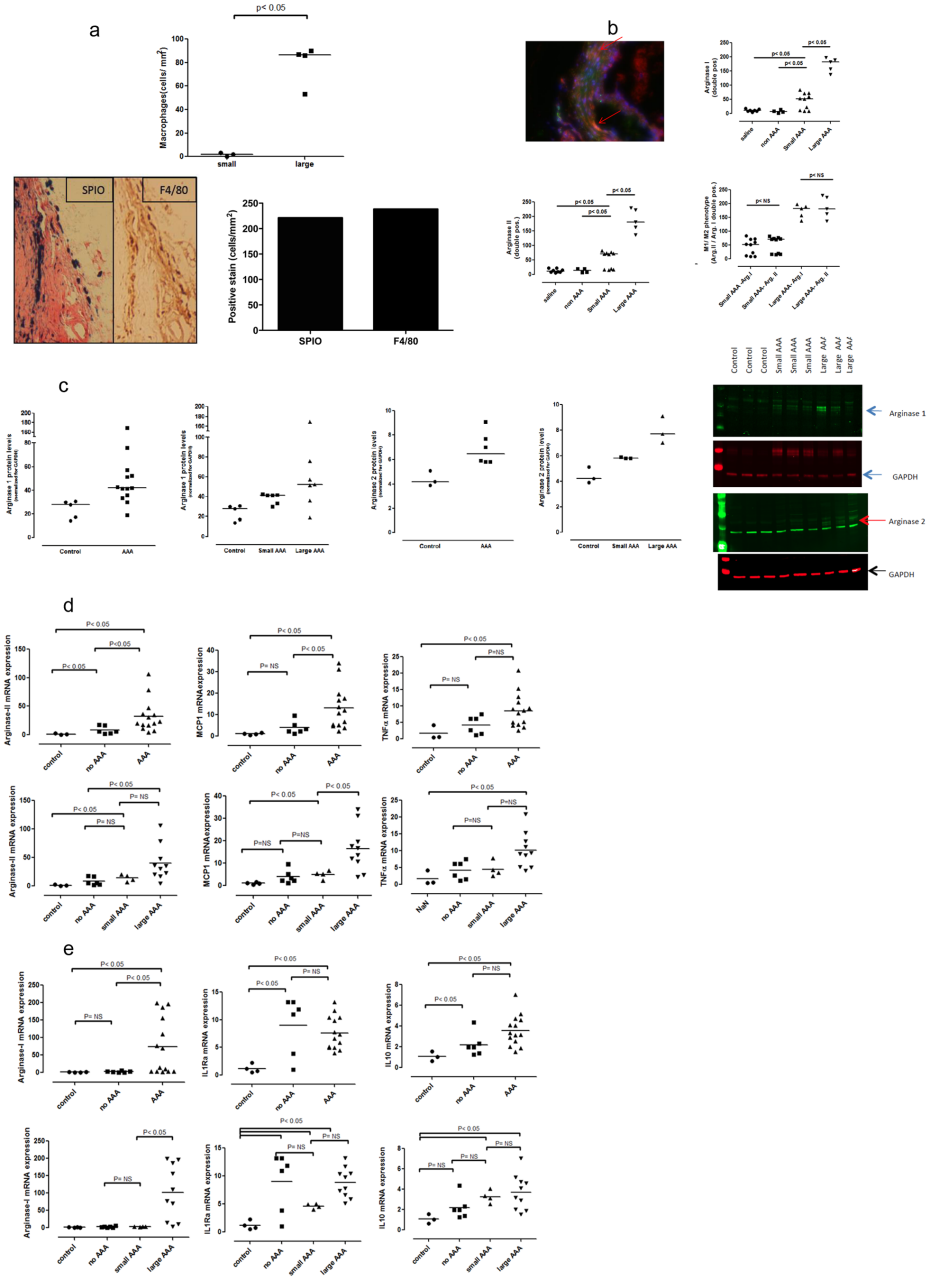
Macrophage expressions in AAA. (A) Analysis of macrophage accumulation in AAA. Prior to sacrifice, mice were injected with SPIO particles via lateral tail vein injections. SPIOs are phagocytosed by macrophages, and are evaluated histologically by Prussian Blue (PB) staining thereby providing an assessment of the presence and activity of macrophages. Top: Analysis using stereology showed a clear increase in positive iron bound macrophages in large aneurysms compared to small aneurysms (small AAA n = 3, large AAA n = 4; p<0.05). Bottom right: Automated quantitative analyses of histological sections of PB and F4/80 stains for macrophages showed congruence in the degree of macrophage accumulation as detected by both methods. Data shown were obtained from analyses of large AAAs; however, similar results were noted for small AAAs (data not shown). Bottom left: Representative histological sections showing SPIO and F4/80 stains for macrophage. Parallel findings from F4/80 and PB stains demonstrates the fidelity of SPIO particles for delineating the presence and activity of macrophages. AAA, abdominal aortic aneurysm; SPIO, supraparamagnetic iron oxide; PB, Prussian Blue. (B) M1 and M2 macrophage phenotypes and AAA. Presence of markers of M1 (arginase II) and M2 (arginase I) in cross sections of normal and aneurysmal tissue was evaluated using immunofluorescence staining (control n = 7, no AAA n = 4, small AAA n = 10 and large AAA n = 5). Increased numbers of positive stains were observed in both small and large aneurysms for both arginase I and arginase II. Expression of arginase I and II were significantly more increased in larger aneurysms. (C) Western blot Analysis of markers of M1 and M2 phenotypes and AAA. Left panels: Protein expression of arginase I as measured by western blot shows a statistically significant increase in arginase I in AAA tissue versus control (control n = 5, AAA n = 13; p p<0.05). Expression of arginase I was higher in large AAA versus small AAA. The observed difference was statistically significant (small AAA n = 6, large AAA n = 7; p<0.05). Middle panels: Protein expression of arginase II as measured by western blot shows small but statistically significant increases of arginase II in AAA tissue versus control (control n = 3, AAA n = 6; p<0.05). Expression of arginase II shows statistically significant differences between control, small and large AAA (small AAA n = 3, large AAA n = 3; p<0.05). Right panel: Representative images of western blot for arginase I and II. (D) Gene expression analysis of markers of M1 phenotype and AAA Left panel: Arginase II gene expression is significantly increased in tissues from both AAA (>23 fold, n = 14) and no AAA (>6 fold, n = 6) mice versus controls (n = 3). The difference between mice with and without AAA (>3 fold) is statistically different. In both small (n = 4) and large AAA (n = 10) the expression of arginase II is significantly different compared to control; however differences between small/large and small/no AAA were not significant. Middle panel: AAA mice had increased expression of MCP1 (>9 fold) compared to those without AAA and controls; both small and large AAA mice had increased expression of MCP1 (p<0.05) compared to controls, and the difference between small and large was significant (p<0.05; control n = 3, no AAA n = 6, small AAA n = 4 and large AAA n = 10). Right panel: TNF-α expression was significantly increased in the presence of AAA (>7 fold increase versus controls). Although both small and large AAA show increased expression (small >4 fold, n = 4, large >9 fold) only the latter differed significantly from controls. Observed differences between large and small AAA were not statistically significant (control n = 3, no AAA n = 6, small AAA n = 4 and large AAA n = 10). (E) Gene expression analysis of markers of M2 phenotype and AAA Left panel: When the size of aneurysm is considered we observed that arginase I expression in small AAA did not significantly differ from that of control but did differ significantly from the expression in large AAA (control n = 3, no AAA n = 6, small AAA n = 4 and large AAA n = 10). Middle panel; IL1Ra gene expression is increased significantly, >6 fold in both AAA and non AAA versus controls. Differences observed between small and large AAA were not significant (control n = 3, no AAA n = 6, small AAA n = 4 and large AAA n = 10). Right panel: Compared to mice in the control group, the IL10 gene expression is significantly increased in mice with AAA (>3 fold). Both small and large AAA differed significantly from control, however differences between small and large were not statistically significant (control n = 3, no AAA n = 6, small AAA n = 4 and large AAA n = 10).

The accrual of large number of macrophages was associated with the development of large AAA (p<0.05) (Figure 5a, top). Presence of SPIO particles in the AAA region was detected by the positive PB stain for iron oxide particles; for SPIOs to be detected they have to be phagocytosed by macrophages, which infers their presence and activity at the site of AAA lesion. The observed differences in degree of macrophage accumulation between small and large AAA was also noted from F4/80 antibody staining of arterial macrophages. Automated quantitative (stereology) analyses of histological sections of PB stain for macrophage-ingested SPIO particles and F4/80 antibody stain demonstrate congruence in the quantity of macrophage accumulation by both methods (Figure 5a, bottom).

Figures 5b to 5e illustrate markers of M1 and M2 macrophages. Immunofluorescence (Figure 5b) demonstrated increased protein expression of markers of both M1 (arginase II) and M2 (arginase I) macrophages in aneurysmal tissue (p<0.05), which was greater in large versus small AAA (p<0.05). Although the pro-inflammatory phenotype would be expected to be at play in the development of AAA, the phenotypic markers did not differ in their relative presence (Figure 5b). This finding was supported by Western Blot analysis, which demonstrated that arginase II and I were statistically significant in AAA tissue versus control (p<0.05) and large versus small AAA (p<0.05)(Figure 5c). Aneurysmal tissues had increased expression of arginase II and MCP1 mRNA (another MI phenotype marker), but only MCP1 expression differed statistically by AAA size (Figure 5d). Arginase I mRNA expression differed by presence AAA size, but other M2 markers (IL1Ra and IL10) did not differ by AAA size (Figure 5e).

### Elastin, Smooth Muscles, and MMP

**Figure 6:**
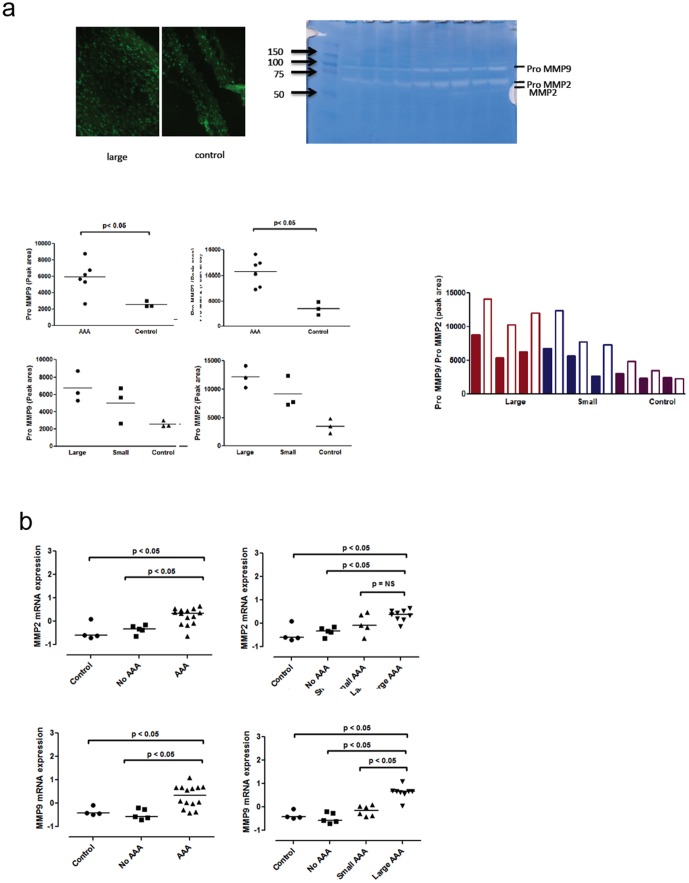
MMP expression in AAA. (A)Top left) In situ zymography shows an increase in gelatin degrading enzymes (MMPs) in large AAA versus control (data for small AAA was similar; data not shown). Top right) Zymography of tissue from both AAA and control aorta revealed the presence of pro-MMP9 and pro MMP2 (and to a minimal extent MMP2). Graphs demonstrate significant differences in peak area analysis between AAA and control. Differences between large and small AAA were not significant but do show a clear trend of increased activity of pro MMP's in large versus small AAA (control n = 3, small AAA n = 3 and large AAA n = 3). (B) Analysis of MMP2 (control n = 4, no AAA n = 5, small AAA n = 5 and large AAA n = 9) and MMP9 (control n = 4, no AAA n = 5, small AAA n = 6 and large AAA n = 8) gene expression demonstrated increased expression of MMPs according the presence of AAA. However, only MMP9 showed increased expression according AAA size.

Consistent with prior reports, immunohistological evaluation demonstrated elastin disruption, medial accumulation of smooth muscle cells in both large and small AAA as previously reported [9]. The degree of elastin disruption did not relate to AAA size. Regarding the presence of MMP in both large and small AAA, a significant difference between AAA and control was noted with respect to MMP9 and pro MMP2. While differences between large and small AAA were not statistically significant, they showed a trend of increased activity of pro MMP's in large versus small AAA (Figure 6a). Gene expression data suggested increased expression of both MMP2 and MPP9 in AAA versus control, and of MMP9 only between small and large AAA (Figure 6b). Western blot analyses confirmed significantly higher MMP9 protein levels of MMP9, in AAA vs. control, but no significant differences between small and large AAA (data not shown).

## Discussion

In this evaluation of the evolution of AngII-induced AAA in mice, we provide the first formal estimates of the odds of AAA occurrence vis-à-vis time, and report a consistent pattern of AAA development. In this model, the aneurysms occur in the first (large AAA) and second (small AAA) weeks of AngII infusion. The odds of occurrence diminished thereafter, and there was no evidence of quantitative regression or change in AAA size following cessation of the aneurysmal stimulus. The size of AAA induced by AngII was not associated with systemic hemodynamic parameters. Macrophage phenotypes were equally represented in AAA development, increasing incrementally with AAA size. Our findings of increased LDL cholesterol levels in mice forming large AAA suggest that the pattern of hypercholesterolemia is associated with AAA size. It must be noted that LDL cholesterol is not the largest component of total cholesterol in the mouse model studied. The effect of small differences in LDL elevation in the context of a massive accumulation of apoB-containing lipoproteins suggests a specific role for this lipoprotein in AAA expansion. These findings are also supported by higher serum PCSK9 levels seen in mice with large AAA. Higher levels of PCSK9 cause lower levels of LDLR and higher levels of LDL. Based on our recent work on reciprocal regulation between PCSK9 and LDLR [12], PCSK9 levels are a means to investigate non-genetic contributions to the unexpected differences in cholesterol levels seen in these genetically identical mice exposed to the same environment. The fact that PCSK9 levels are also high in controls does not diminish the insight that this factor can be at play in the AngII groups.

Collectively, these findings may inform experimental strategies for AAA evaluation, and have potential clinical relevance for risk assessment in AAA patients.

The pathobiology of AAA embodies extracellular matrix degeneration and inflammation as its two major arms, which implicate a complex interplay of key mechanisms that include oxidative stress [13]–[15], local production of proinflammatory cytokines [16]–[17], vascular smooth muscle migration and proliferation [18]–[20], and activation of the three major protease families: matrix metalloproteinases, cysteine, and serine proteases [21]–[26]. Cumulative evidence indicates that the presence and action of smooth muscle cells is essential for Ang II-induced AAA formation and progression [15], [26]–[27]. Similarly, convergent lines of experimental and pathological evidence implicate early involvement of the monocyte/macrophage innate immune response, with consequent production of various proinflammatory cytokines during AAA development [22], [28]–[30].

Of note, it has not been established whether the degree of arterial wall inflammation dictates AAA progression or is simply a consequence of the degenerative process [21]. Recent examination of the kinetics of monocyte recruitment to mouse atherosclerotic lesions suggests that monocyte entry is not confined to the initiation of atherosclerosis, but is progressive and proportional to the extent of disease [31]. In this regard, the striking immunohistological differences associated with diversity and evolution of aneurysms observed in this study add to the body of evidence implicating smooth muscle cells and macrophages in the pathobiology of AAA.

However, the trigger for the differential accrual of macrophages and smooth muscle cells leading to the variation in size and evolution of AAA in congenic mice subjected to the same experimental conditions is intriguing. In this context, our finding that the extent of hypercholesterolemia is an independent predictor of change in aortic diameter evokes the potential paradigm that high cholesterol is a substrate for the accumulation of macrophages, which in the setting of an aneurysmal stimulus (AngII) triggers the cascade of events that leads to AAA development. Thus, increased levels of cholesterol, in the context of a wider accumulation of apoB-containing lipoproteins, may be regulating the degree of macrophage accumulation and setting the trajectory for size and evolution of AAA under the influence of AngII. The mechanisms by which genetically similar mice exposed to the same experimental conditions and stimuli produce different biochemical and vascular responses remain to be elucidated.

The novel paradigm presented here has potential clinical relevance given the quest to mitigate the incidence and progression of AAA, and suggests that the combination of statin and agents blocking angiotensin action should be warranted in all subjects at risk for AAA

Regarding the relationship between serum total cholesterol and final AAA size, it is important to note that we do not contend that baseline or final cholesterol levels dictate the pattern of temporal evolution; rather they are predictive of final AAA size or change in aortic diameter (that is final minus baseline), as the statistical models indicate. However, one must keep in mind that these are genetically identical mice with the same gene defect causing hypercholesterolemia, eating the same high-fat diet, and exposed to the same AngII insult. The finding of any biochemical differences between groups is in itself remarkable. The fact that this difference is exclusively due to LDL, in the context of a much more complex hypercholesterolemic picture, is highly suggestive of a pathogenetic association likely to be causal. Thus we claim that final cholesterol level is predictive of AAA size given the context of controlled experimental design. We believe that such position is supported by the data based on two modeling approaches using as dependent variables either final cholesterol or the difference between baseline and final cholesterol. Although there was a temporal effect, we do not ascribe it to changes in cholesterol level given the absence of serial lipid measurements during the study period.

### Limitations

A major part of this study rests on the in vivo US evaluation and measurements of mouse aorta, thus raising questions about the reliability of measurements derived from this modality. However, these systems have been reported to have 100% accuracy for detecting AAA, and the quantitative estimates of precision are on the order of 0.1 to 0.01 mm in AAA and non-AAA regions regardless of variations in AAA size [9], [32]–[33].

In addition, although AAA development was independent from blood pressure, the latter was measured only via tail cuff method, a non-invasive approach that is inferior to invasive assessment of hemodynamics. However, despite the noninvasive nature of the tail cuff method of BP assessments, reliability of data was increased by repeat measurements (average of 10 readings per mouse at each time point). Furthermore, our findings that BP is of no relevance to AAA development is consistent with that reported by all other groups.

## Conclusions

We have shown that in the mouse model of AngII-induced AAA there is a differential response to the aneurysmal effects of AngII as indicated by the variation in the size and time of AAA occurrence, and that the degree of macrophage accrual may be relevant to the observed differential response to AngII, with the extent of hypercholesterolemia as the potential underpinning factor. The mechanisms that underlie these findings may improve our understanding of the pathobiology of AAA, and should inform more targeted investigations.

## Supporting Information

Figure S1 **Systemic Hemodynamics and AAA development.** (A) Left panel, serial hemodynamic assessment demonstrates an increase in diastolic blood pressure following Ang II infusion, which plateaued in the second week of infusion. However, among mice exposed to Ang II there were no differences in diastolic blood pressure between those that developed AAA and those that did not. Right panel, similar diastolic blood pressure pattern was noted independent of the size of AAA. Findings from the analysis of mean arterial blood pressure (B) according to occurrence and size of AAA were consistent with those of diastolic pressure assessment. Serial assessments of pulse rate (C) and pulse pressure (D) revealed variability and overlap between Ang II exposed and control mice regardless of the occurrence or size of AAA. Overall, statistical comparison revealed no significant difference in all hemodynamic parameters between mice that developed AAA versus those that did not; likewise, there was no difference by AAA size.
(TIF)

Methods S1 **Supplementary methods.** discribing details on tail vein injection, hemodynamic assessment, atherosclerotic lesion quantification, quantitative gene expression, analyses of serum total cholesterol, lipoproteins, and PCSK9, zymography for metalloproteinases, and immuno-histology and fluorescence analysis.
(DOCX)
