# Supplementary figures and images for: Angiotensin-Induced Abdominal Aortic Aneurysms in Hypercholesterolemic Mice: Role of Serum Cholesterol and Temporal Effects of Exposure
Source: PLoS One. 2014 Jan 23;9(1):e84517. doi: 10.1371/journal.pone.0084517 (PMC3900396; doi:10.1371/journal.pone.0084517)

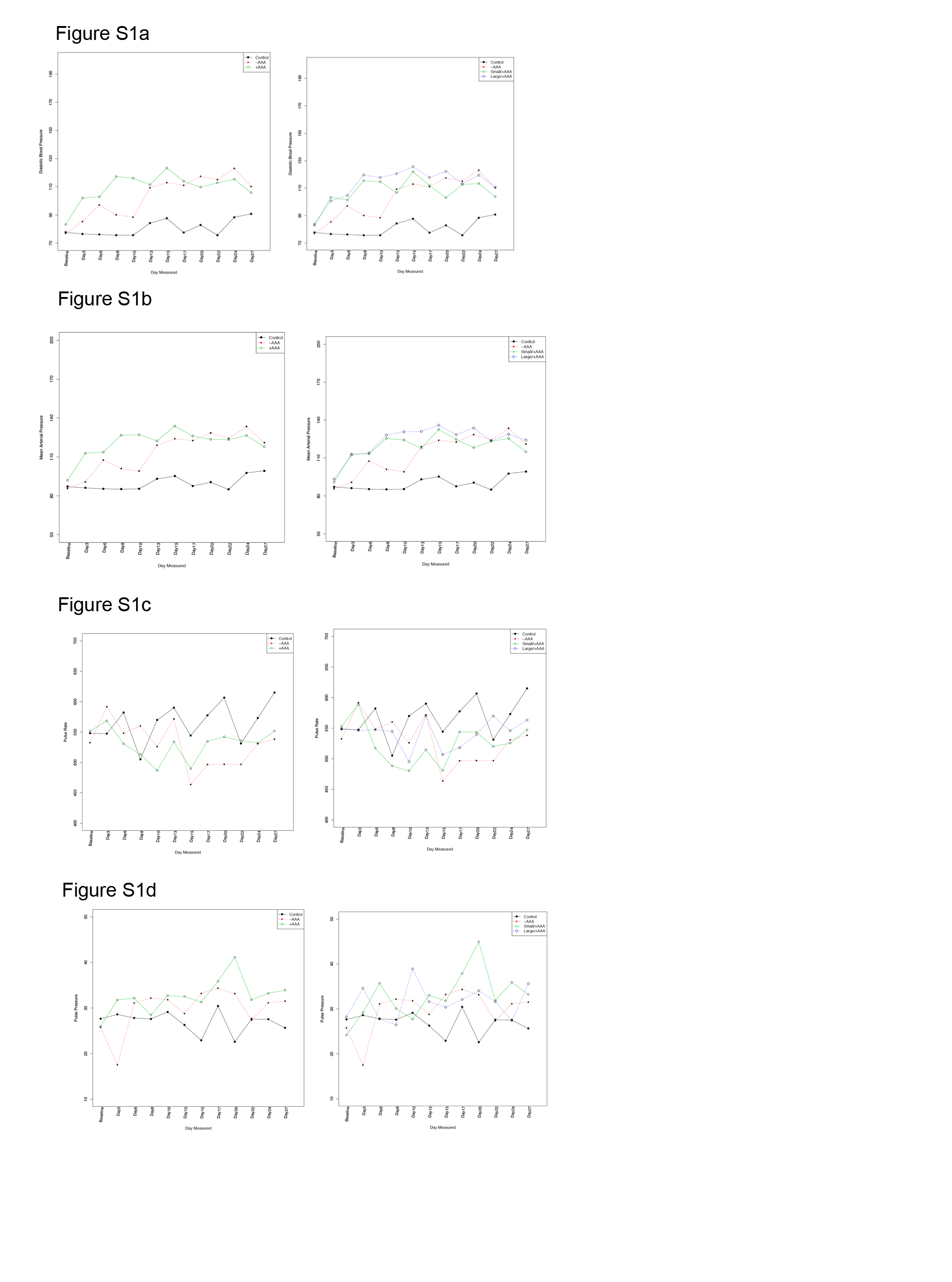

Supplement: Figure S1 — Systemic Hemodynamics and AAA development. (A) Left panel, serial hemodynamic assessment demonstrates an increase in diastolic blood pressure following Ang II infusion, which plateaued in the second week of infusion. However, among mice exposed to Ang II there were no differences in diastolic blood pressure between those that developed AAA and those that did not. Right panel, similar diastolic blood pressure pattern was noted independent of the size of AAA. Findings from the analysis of mean arterial blood pressure (B) according to occurrence and size of AAA were consistent with those of diastolic pressure assessment. Serial assessments of pulse rate (C) and pulse pressure (D) revealed variability and overlap between Ang II exposed and control mice regardless of the occurrence or size of AAA. Overall, statistical comparison revealed no significant difference in all hemodynamic parameters between mice that developed AAA versus those that did not; likewise, there was no difference by AAA size. (TIF) [file pone.0084517.s001.tif]
