# Supplementary material for: Angiotensin-Induced Abdominal Aortic Aneurysms in Hypercholesterolemic Mice: Role of Serum Cholesterol and Temporal Effects of Exposure
Source: PLoS One. 2014 Jan 23;9(1):e84517. doi: 10.1371/journal.pone.0084517 (PMC3900396; doi:10.1371/journal.pone.0084517)
Supplement: Methods S1 — Supplementary methods. discribing details on tail vein injection, hemodynamic assessment, atherosclerotic lesion quantification, quantitative gene expression, analyses of serum total cholesterol, lipoproteins, and PCSK9, zymography for metalloproteinases, and immuno-histology and fluorescence analysis. (DOCX) [file pone.0084517.s002.docx]

*Tail vein injection of supraparamagnetic iron oxide (SPIO) particles*

Prior to sacrifice, a small group of mice (n = 10) were injected with SPIO particles[^11^](#_ENREF_11) (10 mg/kg) via lateral tail vein injections. SPIOs are phagocytosed by macrophages, and are evaluated histologically by Prussian Blue (PB) staining thereby providing an assessment of the presence and activity of macrophages.

*Hemodynamic assessments*

Fifty-nine mice underwent hemodynamic assessment for determination of systolic, diastolic, and mean arterial pressures (MAP), and pulse rate and pulse pressure using a BP-2000 series II apparatus BP 2000 Specimen Platform, Visitech Systems, North Carolina). Measurements were performed with a tail cuff set up.

*Atherosclerotic lesion area quantification*

Mice were sacrificed and the aorta was flushed with cold PBS by injection through the left ventricle. Heart and aortic tissue was then embedded in OCT and flash frozen in liquid nitrogen. Frozen sections of 10µm thickness were cut from the aortic sinus, according to standard procedure. Sections were stained with Oil Red O and counterstained with hematoxylin. Quantification of lesion area was performed on 15 sections per mouse (n=33) and is reported in mean µm^2^/section. Images were captured with a frame grabber (Vers3.1 Kontron Electronik), using an Axiokop microscope with mounted camera (Carl Zeiss Inc). The imaging system KS300 (Kontron Electronik) was used for quantitative analysis of lesion areas.

*Quantitative gene expression*

Frozen aortas were homogenized using a mortar and pestle in the presence of nitrogen. Total RNA was then extracted by means of Trizol (Sigma, USA). RNA was further isolated, cleaned and DNase treated using RNeasy spin columns (Qaigen, USA) according to manufacturers protocol. RNA quality was assessed by determining 28S and 18S ribosomal RNA integrity using nanoprobe and Bioanalyzer (Bio-rad, USA). 1ug of total RNA was then reverse transcribed to cDNA using the iScript cDNA Synthesis Kit (Bio-rad, USA). In short, 5xiScript reaction mix was mixed with reverse transcriptase, nuclease free H2O and RNA template. The mixture was incubated for 5 min at 25^o^C, 30 min at 42^o^C, 5 min at 85 ^o^C, and held at 4^o^C until further use. 20 ng of the resulting cDNA was then used to quantitatively measure the expression of genes using SsoFast Evagreen Supermix (Bio-Rad, USA) according to manufacturer’s protocol using the following cycling conditions; activation for 30 sec. at 95 ^o^C, denaturation for 5 sec. at 95 ^o^C, and annealing/ extension for 5 sec. at 60 ^o^C. The primers that were used in gene expression are found in the box insert. Real time measurements were performed with a Bio-Rad CFX96 Real Time PCR System and analyzed using q-Base plus software (Biogazelle).

| Box Insert | |
| --- | --- |
| Gene | Primer sequence |
| Arginase I | 5’-GAACACGGCAGTGGCTTTAAC-3’  5’-TGCTTAGCTCTGTCTGCTTTGC-3’ |
| Arginase II | 5’-TGATTGGCAAAAGGCAGAGG-3’  5’-CTAGGAGTAGGAAGGTGGTC-3’ |
| IL10 | 5’-GAAGACCCTCAGGATGCG-3’  5’-CAAAGGAGTTGTTTCCGTTA-3’ |
| IL1RA | 5’-ACAGTAGAAGGAGACAGAAG-3’  5’-GGTGGTAGAGCAGAAGAC-3’ |
| MCP1 | 5’-GCTCAGCCAGATGCAGTTAAC-3’  5’-CTCTCTCTTGAGCTTGGTGAC-3’ |
| MMP2 | 5’-ACTCCAGTTAAAGGCAGCATCTAC-3’  5’-CAGGGAATGAGTACTGGGTCTATT-3’ |
| MMP9 | 5’-AGCTGATTGACTAAAGTAGCTGGA-3’  5’-AATCTCTTCTAGAGACTGGGAAGGAG-3’ |
| TNF-α | 5’-GTGGAACTGGCAGAAGAG-3’  5’-CCATAGAACTGATGAGAGG-3’ |
| 18S | 5’-GAATGGTGCTACCGGTCATT-3’  5’-ACCTCTCTTACCCGCTCTCC-3’ |
| GAPDH | 5’-GGCCTTCCGTGTTCCTACC-3’  5’-CGGCATGTCAGATCCACAAC-3’ |
| EF-1 | 5’-GCAAGCCCATGTGTGTTGAA-3’  5’-TGATGACACCCACAGCAACTG-3’ |

*Quantitative protein expression*

Snap frozen tissue was homogenized under presence of nitrogen and lysed using RIPA buffer supplemented with protease inhibitors (Pierce, USA) for 45 minutes on ice. Protein concentrations of the whole cell lysate are determined using the Bradford Protein Assay ( Biorad, USA). Total cell lysates are separated on SDS-Page using electrophoresis, followed by electro transfer into nitro cellulose membranes (Millipore, USA). Membranes are blocked for one hour in Odyssey blocking buffer (Licor, USA) followed by incubation with primary (Arginase 1 and arginase 2, Genetech, USA) and secondary antibodies. The Licor/ Odyssey scanner and software was used for detection and signal and density evaluation.

*Analyses of serum total cholesterol, lipoproteins, and PCSK9.*

Blood samples were collected from 65 mice prior to start of treatment and prior to sacrifice to determine baseline and final concentrations of total cholesterol. Cholesterol levels were determined using Raichem reagent #80015VI and Sigma Reagent No.339 adapted for microtiter plate assay. Mouse lipoproteins were prepared by fast protein liquid chromatography (FPLC) analysis of serum using a Superose 6 column (Pharmacia) on a FPLC system with a Waters 500 pump. Aliquots of individual or pooled mouse serum (100 ul) were injected onto the column and separated with a buffer containing 0.15 M NaCl, 0.01 M Na2HPO4, 0.1 mM EDTA, at a flow rate of 0.5 ml/min. Forty fractions of 0.5 ml each were collected, with lipoproteins eluting within fractions 14-33 as follows: 14-19 VLDL, 20-25 LDL and large HDL, 26-33 HDL. Cholesterol levels were determined using the same reagent described above.The levels of murine PCSK9 were measured in diluted serum (1:100) of fasting mice and determined by ELISA kits (MBL-International), according to the manufacturer instructions and within the kit’s linear range.

*Zymography for metalloproteinases*

Novex Zymogram Gels (Invitrogen, USA) were used to detect MMP9 and MMP2 according to manufactures guidelines. In short: samples were denatured in SDS buffer under non-reducing conditions and without heating, and run on a zymography gel using Tris-Glycine SDS Running Buffer.  Upon completion gels are incubated in Zymogram Renaturing buffer.. The gels are then equilibrated in Zymogram Developing Buffer prior to staining them using Chromassie Blue staining.

*Immunohistology*

Immunohistochemical staining was performed to assess the presence of macrophages, elastin degradation, and vascular smooth muscle cells. Embedded tissue were cut into 3-micron thick serial sections prior to being stained with Weigert’s Iron Hematoxylin for 15 minutes and Elastin van Gieson for 5 minutes. Slides were incubated with primary antibody at room temperature for 1 hour (F4/80, (macrophages) (GeneTex, CA). Potential non-specific sites were blocked using normal serum for 30 minutes. Negative controls were prepared without primary antibody to check for non-specific staining. Anti-Mouse, Anti-Rat and Anti-Rabbit Vectastain Elite ABC kits (Vector Laboratories, CA) were used for the secondary biotinylated antibody and streptavidin-peroxidase complex. A Vector Nova Red substrate kit (Vector Laboratories, CA) was used as substrate for the peroxidase; if counterstained, Gills Hematoxylin III (EMD, USA) was used. Slides were simultaneously stained and quantified by two investigators. A vanguard 1490-FLP01 (VeeGee Scientific, WA) microscope with mounted camera (Canon Eos Rebel xsi) was used for analysis of slides and image capture. Automated analyses of stains for macrophages from biological replicates were done by stereology. For this, a microscope with mounted camera (Olympus BX50W1) and stereology software (stereologer 2000) was used.

*Fluorescence staining of macrophages*

The abdominal part of the aorta containing the aneurysm was embedded in OCT and frozen. Serial cryosections, 5-μm thick, were cut and fixed in acetone prior to an overnight incubation with a M1/ M2 macrophage phenotype specific antibody (Arginase I, Genetex for phenotype II and Arginase II, Protein Tech, for phenotype I) (1:50), whereas biotinylated Lamp-2 and nuclear DAPI were used to stain macrophages. Goat anti Rabbit (Texas Red) and Streptavidin (FITC) were used as secondary antibodies. M1/ M2 expression was assessed based on double positive stain.
